# Evolutionary lability of a complex life cycle in the aphid genus *Brachycaudus*

**DOI:** 10.1186/1471-2148-10-295

**Published:** 2010-09-28

**Authors:** Jousselin Emmanuelle, Genson Gwenaelle, Coeur d'acier Armelle

**Affiliations:** 1 INRA, UMR CBGP (INRA/IRD/Cirad/Montpellier SupAgro), Campus international de Baillarguet, CS 30016, F-34988 Montferrier-sur-Lez cedex, France

## Abstract

**Background:**

Most aphid species complete their life cycle on the same set of host-plant species, but some (heteroecious species) alternate between different hosts, migrating from primary (woody) to secondary (herbaceous) host plants. The evolutionary processes behind the evolution of this complex life cycle have often been debated. One widely accepted scenario is that heteroecy evolved from monoecy on woody host plants. Several shifts towards monoecy on herbaceous plants have subsequently occurred and resulted in the radiation of aphids. Host alternation would have persisted in some cases due to developmental constraints preventing aphids from shifting their entire life cycle to herbaceous hosts (which are thought to be more favourable). According to this scenario, if aphids lose their primary host during evolution they should not regain it. The genus *Brachycaudus *includes species with all the types of life cycle (monoecy on woody plants, heteroecy, monoecy on herbs). We used this genus to test hypotheses concerning the evolution of life cycles in aphids.

**Results:**

Phylogenetic investigation and character reconstruction suggest that life cycle is evolutionary labile in the genus. Though ancestral character states can be ambiguous depending on optimization methods, all analyses suggest that transitions from monoecy on herbs towards heteroecy have occurred several times. Transitions from heteroecy towards monoecy, are also likely. There have been many shifts in feeding behaviour but we found no significant correlation between life cycle changes and changes in diet.

**Conclusions:**

The transitions from monoecy on herbs towards heteroecy observed in this study go against a widely accepted evolutionary scenario: aphids in the genus *Brachycaudus *seem to be able to recapture their supposedly ancestral woody host. This suggests that the determinants of host alternation are probably not as complicated as previously thought. Definitive proofs of the lability of life cycle in *Brachycaudus *will necessitate investigation of these determinants. Life cycle changes, whether corresponding to the loss or acquisition of a primary host, necessarily promote speciation, by inducing shifts of the reproductive phase on different plants. We suggest that the evolutionary lability of life cycle may have driven speciation events in the *Brachycaudus *genus.

## Background

Parasitic organisms, whether bacteria, viruses, fungi, nematodes or phytophagous insects, sometimes make use of a sequence of different hosts during their life cycle. Such host alternation is so common among parasites that many courses in parasitology are devoted largely to descriptions of parasitic life cycle and the sequences of host changes. The evolution of such complex life cycle is puzzling in many ways. In particular, parasites displaying host alternation may show extreme specificity and adaptations for two very distantly related hosts with few features in common [1]. Several questions have been raised about the evolution of host alternation. Is host alternation evolutionarily labile or is it a rare evolutionary change in various phyla? Is it adaptive and, if so, what advantages does it confer on parasites with this trait? What effect does it have on parasite diversification? Do complex life cycles favour the colonization of new ecological niches and enhance cladogenesis?

Such questions can be addressed in aphids (Hemiptera, Aphididae, *sensu stricto *[2]). Aphids and their closest relatives, the Phylloxeridae and Adelgidae, are the only phytophagous insects, with cynipid wasps [3], known sometimes to use two sets of host plants during their life cycle. These species are described as "heteroecious" or as displaying "host alternation" [4]. Host alternation is found in about 10% of the 4500 described aphid species, the other 90% of aphids using the same group of host plants throughout their entire life cycle (these species are described as monoecious). In most heteroecious Aphididae species, the cycle follows a similar pattern: the eggs hatch and colonies develop parthenogenetically on their primary/winter hosts (mostly woody hosts); the aphids then migrate to secondary/summer hosts (mostly herbaceous plants) on which they reproduce parthenogenetically; subsequent generations then return to the primary hosts for sexual reproduction and overwintering. In most cases the alternate sets of hosts are not taxonomically related and have no obvious ecological similarities [5]. However, life cycles involving host alternation are thought to reflect the order in which plant taxa were acquired as host plants by aphid lineages. Monoecy on trees is assumed to be the ancestral state in the family [6-8], with host alternation being acquired at the time of initial grass diversification (late Cretaceous), followed in some cases by the loss of the primary host tree species and monoecy on grasses [9,10]. The evolution of the aphid life cycle, the selective pressures driving it and its consequences for aphid diversification have been a matter for lively debate over many years.

Heteroecy is observed in different aphid subfamilies and the means of returning to primary hosts depend on the subfamily concerned, strongly suggesting that several acquisitions of this trait have occurred in the Aphididae [7,10,11]. The most recent phylogenetic study suggests a minimum of two independent acquisitions of host alternation [12]. Multiple acquisitions of host alternation have also been suggested in the aphid subfamily with the largest number of species, the Aphidinae, as many genera in this subfamily contain both heteroecious and monoecious species [13,14]. It is difficult to infer the exact number of gains and losses of heteroecy in aphids based on current reconstructions: the phylogenies of Aphididae, or even Aphidinae [15], are still largely unresolved, probably because aphids have undergone rapid radiation [10,12]. The evolutionary forces driving the evolution of this trait are also poorly understood. There are two points of view concerning the evolution of host alternation in aphids [7,8,16]. The first view suggests that having two hosts is adaptive [4,17], as it allows the optimal exploitation of the available plants. Numerous adaptive advantages have been proposed (nutrient availability, enemy escape etc.) [7,8], but all arguments boil down to the general idea that aphids use two sets of hosts when this is optimal for their growth and parthenogenetic reproduction. Another point of view is that this trait results from developmental constraints affecting several aphid morphotypes [7,18]. According to this hypothesis, the use of two hosts is not necessarily advantageous and primary hosts are retained because of strong phylogenetic constraints on the morph that hatches from overwintering eggs in spring, the fundatrix. This morph is highly adapted to the primary hosts, and less able to switch hosts than other morphs. The oviposition preferences of the sexual morphs for the woody host are also likely to be subject to evolutionary constraints. This would imply that the loss of the primary host and the shift of the entire cycle to a new secondary herbaceous host requires an escape from both these constraints, entailing several major, simultaneous mutations [19]. This hypothesis, known as "the fundatrix specialisation hypothesis", generates a straightforward prediction: if aphids manage to lose their primary host during evolution, thus escaping from the dead-end resulting from fundatrix specialisation, then a return to heteroecy should not occur [19]. Hence, monoecy on herbaceous hosts, which occurs in several Aphidinae genera, should always be a derived state. Finally, the evolution of host alternation is also often described as having played an important role in favouring the colonisation of new plant species and speciation in aphids. Indeed, the acquisition of heteroecy may be followed by a shift of all or part of the population to permanent existence on some of the new secondary hosts and, therefore, by speciation [10]. The species-rich genera of the Aphidinae that are monoecious on herbaceous plants are thought to have arisen through the loss of their primary host, followed by extensive radiation on their newly acquired herbaceous host [13,20].

In this study, we addressed some of the questions concerning the evolution of life cycles in aphids, by focusing on the evolution of this trait in one genus of the Aphidinae subfamily: the genus *Brachycaudus *van der Goot, 1913. This genus has 43 species [2,21], 14 of which always or sometimes spend some of their life cycle on *Prunus *species [22]. There are about 5 heteroecious species associated with secondary host plants belonging to the Asteraceae, Boraginaceae, Caryophyllaceae, Lamiaceae, Polygonaceae and Scrophulariaceae [20]. Other species are monoecious, living either on Rosales or on herbaceous hosts, some of which belong to the same families as the secondary host plants of heteroecious species [14] (see Additional file 1 for details on the biology of *Brachycaudus *species). Hence, this genus displays all the types of life cycle: monoecy on a woody host, host alternation between a woody host and herbaceous hosts, and monoecy on herbaceous hosts. Some species in the genus thus appear to have lost their primary rosaceous hosts to become monoecious on more recently acquired secondary herbaceous hosts (also called secondary monoecy) [9,15]. We investigated this hypothetical scenario through phylogenetic reconstruction of the genus and addressed several questions: 1) What is the ancestral life cycle state in this genus? 2) How many gains and losses of host alternation have occurred in this genus and, once lost, has heteroecy ever been regained? 3) What are the consequences of evolutionary changes in aphid life cycle for the association of these insects with their host plants?

We expand on our previous phylogenetic studies of the genus [23,24], by adding several extra species and specimens to our sampling. We have also added a nuclear DNA fragment, to improve phylogenetic resolution. Our previous studies showed that some of the species of the genus *Brachycaudus *had been ambiguously defined. Some specimens identified as belonging to the same species failed to cluster as a clade and formed polytomies with specimens belonging to very closely related species but defined as having a different life cycle and/or host range. Our objective here was to infer the evolutionary history of life cycle and host association. We therefore ensured that inferences concerning the number of evolutionary transitions in these characters were as conservative as possible, by applying a DNA-based species delimitation method to our dataset. This made it possible to define species independently of the ecological traits we wanted to study.

## Results

### Phylogenetic reconstructions

**Figure 1:**
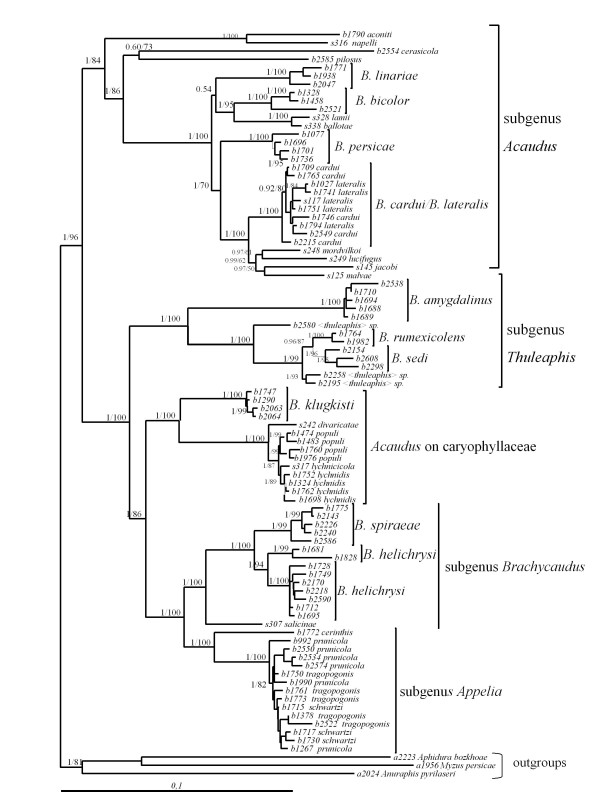
**Phylogenetic reconstruction of *Brachycaudus*. **ML topology and branch lengths are shown. Pp and ML bootstrap values are indicated above nodes. Taxonomic subdivisions (following [2]) are indicated on the left.

We obtained a well resolved phylogenetic tree (Fig. 1), in which most of the nodes were supported by high ML bootstrap and *pp *values. The phylogenetic relationships obtained here were very similar to those reported previously [23,24], and the addition of a nuclear marker confirmed the previously reported phylogenetic relationships between the species sampled. Two additional species from Central Asia, *Brachycaudus pilosus *(Mordvilko, 1929) and *Brachycaudus cerasicola *(Mordvilko, 1929), not included in previous reconstructions, clustered together with relatively long branches as a sister group to the species of the subgenus *Acaudus. *However, support for this branch was poor, suggesting possible long branch attraction [25].

### Species delimitation

**Figure 2:**
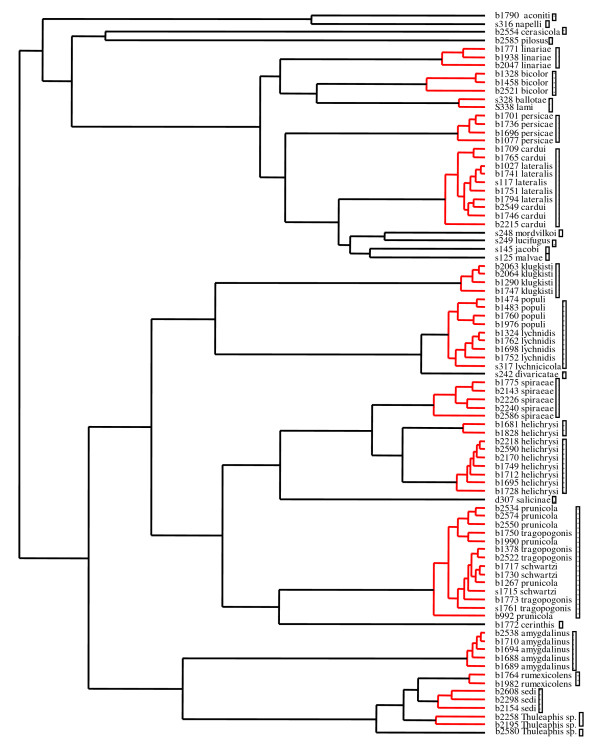
**Species delimitation results. **The vertical bars group all specimens identified as belonging to a significant cluster.

Our sampling initially comprised 29 recognised *Brachycaudus *species identified on the basis of classical taxonomy and three unidentified specimens. For the *Brachycaudus *ultrametric tree obtained with Multidivtime, the GMYC model was preferred over the null model of uniform branching patterns (2ΔL = 19.67, χ_2 _test *P *<< 0.0001). This species delimitation method retrieved 27 phylogenetic species of *Brachycaudus *(Fig. 2). Most species recognized by classical taxonomy were retrieved by our method. However, this method also clustered several species together. *B. lateralis *(Walker, 1848) and *B. cardui *(Linnaeus, 1758) specimens were grouped together as a single entity. This suggests that *B. lateralis *and *B. cardui *do not form the two species generally defined -- one mostly anholocyclic (without a sexual phase) on Asteraceae and the other alternating between *Prunus *and plants from several different families (mostly Asteraceae and Boraginaceae) -- but instead form a single species displaying host alternation between *Prunus *and several plant families. *B. lateralis *is indeed sometimes treated as a subspecies of *B. cardui*. All species of the subgenus *Appelia *other than *B. cerinthis *also clustered together, even if several specimens from central Asia were included. *Brachycaudus *(*Acaudus*) spp. associated with Caryophyllaceae, except *B. divaricatae *(Shaposhnikov, 1956) and *B. klugkisti *(Börner, 1942), also formed a single genetic cluster. Specimens identified as *B. lami *(Koch, 1854) and *B. ballotae *(Passerini, 1860) were also identified as belonging to a single species. These clustering patterns confirmed our previous findings, based on less extensive specimen and species sampling and fewer DNA markers [23]. The clusters obtained all corresponded to groups of species in which species identification can be difficult and specimens may be assigned to species on the basis of host affiliation or morphological characters subject to intraspecific variation. However, this method detected two clusters within *B. helichrysi *(Kaltenbach, 1843) and two clusters among the *Thuleaphis *specimens collected in Kazakhstan that we failed to identify. Two of the *Thuleaphis *specimens collected on *Atraphaxis sp. *formed a phylogenetic cluster, and one specimen collected on a *Rheum *sp. but morphologically different from *B. rumexicolens *(Patch, 19717) appeared as a separate, differentiated taxon. These two phylogenetic species may correspond to the two new *Thuleaphis *species reported by Kadyrbekov [26,27].

### Character evolution

**Figure 3:**
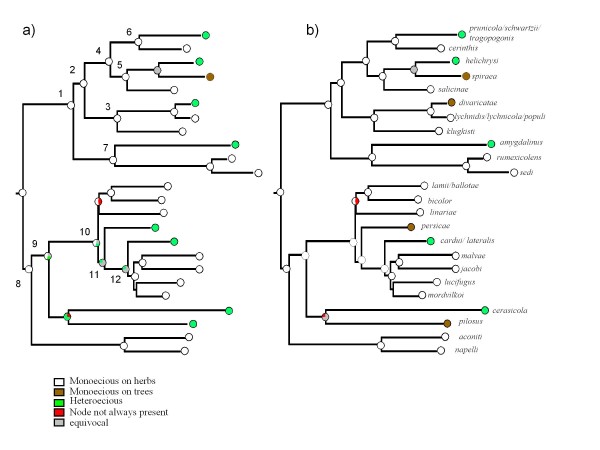
**Life cycle evolution shown on one Bayesian species tree. **Pie charts at nodes show the% of trees for which the character state at this node was identified as the uniquely best state under MP optimisation (the percentage of equivocal reconstructions include reconstructions that did not yield a single best state for the node concerned, even if one state was more likely than any other): (a) life cycle evolution with facultative heteroecious species considered to be heteroecious; (b) life cycle evolution with facultative heteroecious species considered to be monoecious on trees. ML optimization under the 6 parameter model suggested that all character states were equally likely at all nodes of interest (nodes 1 to 12).

**Figure 4:**
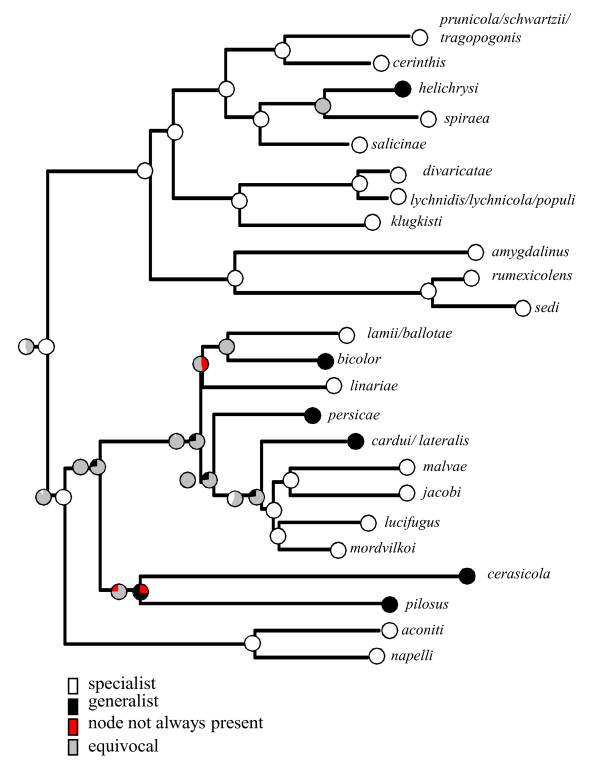
**Host range breadth evolution shown on one Bayesian species tree. **Pie charts at nodes show the % of trees for which the character state at this node was identified as the uniquely best state. ML optimization is given by the first pie chart on the left and MP optimization by the second pie chart

Ancestral state reconstruction by MP, ML and stochastic mapping are summarised in Fig. 3 and 4 and Tables 1-2-3.

**Table 1:** Results of ML investigation of models of rates of change in life cycle and comparisons of reconstructions under "fossilized" character states at several nodes using BayesTraits.

	Model	Description	Likelihood^1^
**A**^**2**^	**6 parameters**	**A different rate for each possible transition**	**-17.90**

	1 parameter	A single transition rate	-23.57

**B**^**2**^	**6 parameters**	**A different rate for each possible transition**	**-20.92**

	1 parameter	A single transition rate	-23.67

A	Q01 and Q02 = 0	No loss of monoecy on herbs (i.e. no capture of woody host)	-22.45

	**Q10 and Q20 = 0**	**No acquisition of monoecy on herbs**	**-19.99**

B	Q01 and Q02 = 0	No loss of monoecy on herbs (i.e. no re-capture of woody host)	-25.55

	**Q10 and Q20 = 0**	**No acquisition of monoecy on herbs**	-22.92

Reconstructions under constraints in node^3 ^states			

A	Node 3, 6, 12 fossilized to 1 (heteroecious)		-21.86

	**Node 3, 6, 12 fossilized to 0 (monoecious on herbs)**		**-19.14**

B	Node 3, 6, 12 fossilized to 1 (heteroecious)		-25.46

	**Node 3, 6, 12 fossilized to 0 (monoecious on herbs)**		**-22.16**

A	Node 1 to 12 fossilized to 1 (heteroecious)		-23.46

	**Node **1 to 12 **fossilized to 0 (monoecious on herbs)**		**-19.99**

B	Node 1 to 12 fossilized to 1 (heteroecious)		-27.55

	**Node 1 to 12 fossilized to 0 (monoecious on herbs)**		**-22.92**

^1^For each analysis, likelihoods given in the table are average likelihoods for the 100 trees (there was very little difference between trees).

^2^Two scenarios were investigated each time: A) facultative heteroecious species considered as heteroecious; B) facultative heteroecious species considered as monoecious on trees.

^3^Node numbers refer to those in Fig. 3.

**Table 2:** Bayesian posterior probabilities for life cycle and host range breadth states estimated with SIMMAP.

	Life cycle						Diet	
**Node**	**Monoecious on herbs**		**Heteroecious**		**Monoecious on trees**		**Specialist**	**Generalist**

	A	B	A	B	A	B		

root	0.99	1.00	0.003	0.00	0.00	0.00	0.99	0.003

1	0.99	1.00	0.002	0.00	0.00	0.00	0.99	0.001

2	1.00	1.00	0.00	0.00	0.00	0.00	1.00	0.00

3	1.00	1.00	0.00	0.00	0.00	0.00	1.00	0.00

4	0.99	0.99	0.01	0.006	0.00	0.00	1.00	0.00

5	0.78	0.78	0.21	0.214	0.005	0.005	1.00	0.00

6	0.63	0.63	0.367	0.367	0.00	0.00	1.00	0.00

7	0.98	0.99	0.017	0.013	0.00	0.00	0.98	0.02

8	0.99	1.00	0.004	0.00	0.00	0.00	0.99	0.005

9	0.58	0.99	0.422	0.003	0.00	0.006	0.67	0.315

10	0.97	0.99	0.03	0.00	0.00	0.001	0.99	0.003

11	0.70	0.99	0.30	0.00	0.00	0.003	1.00	0.00

*12*	0.53	0.98	0.47	0.02	0.00	0.00	0.99	0.002

Node numbers refer to those in Fig. 3. Different prior distributions for the rates of evolution of each character were tested: we show results corresponding to α = 3 and β = 2 (the gamma distribution was discretised into 50 categories) for both characters, as different combination of priors gave very similar results. A: facultative heteroecious species considered heteroecious. B: facultative heteroecious species considered as monoecious on trees.

**Table 3:** Summary statistics for simulated character histories obtained with SIMMAP: estimated number of transitions from one state to another for life cycle are given: state 0 (monoecious life cycle on herbs); state 1 (heteroecious); state 2 (monoecious on woody host), and for feeding diet state 0 (specialist diet); state 1 (generalist).

	Number of transitions	0 = > 1	0 = > 2	1 = > 0	1 = > 2	2 = > 0	2 = > 1
Life cycle A	9.26	5.82	0.46	1.83	0.65	0.04	0.45

Life cycle B	9.75	3.95	3.26	0.67	0.78	0.12	0.97

Diet		0 = > 1	1 = > 0				

	5.73	5.34	0.33		-	-	-

Different prior distributions for the rates of evolution were tested: we show results corresponding to the same priors than Table 2. A: facultative heteroecious species considered as heteroecious; B: facultative heteroecious species considered as monoecious on trees.

The use of different prior distributions for character rates of evolution for stochastic mapping had no effect on the results obtained. MP and stochastic mapping both suggested that, based on our species sampling, the most likely ancestral state in the genus *Brachycaudu*s was monoecy on herbaceous plants, regardless of whether facultatively heteroecious species were considered to be heteroecious or monoecious on trees. For ML reconstructions, a full model (using 6 transition rates, one for each type of change) was preferred over a single rate model or simplified models where some transition rates were set to zero (see Table 1 for details). Using this 6 parameter model yielded uncertainty at the root of the tree with no particular state identified as best state (Fig. 3).

Character states reconstructions suggested that there have been numerous transitions in life cycle (Fig. 3, Tables 1-2-3). MP reconstruction, which suggested that there have been eight or nine life cycle transitions during the evolution of the genus, inferred that there had been four or five (re-)acquisitions of heteroecy from monoecy on herbaceous plants, depending on the status assigned to facultatively heteroecious species. It identified at least five (re-)acquisitions of woody hosts (one in each of the following subgenera: *Appelia*, *Thuleaphis*, *Brachycaudus*, one in the group of species associated with Caryophyllaceae, and at least one in the subgenus *Acaudus*. MP reconstructions suggested that two additional life cycle transitions have occurred in the *Acaudus *subgenus, which were equally likely to be gains or losses of host alternation. Ancestral states were equivocal at all nodes when the results of ML reconstructions were summarised from the sampled 100 trees (Fig. 3). However, the model setting transition rates from state zero (monoecious on herbs) towards states 1 (heteroecious) and 2 (monoecious on trees) to zero, was significantly worse than a model without any constraint or a model setting the reverse transitions to zero (Table 1). Further, when character states at several nodes of interest (nodes clustering species with contrasted life cycle states, e.g. species in the subgenus *Appelia*, species associated with Caryophyllaceae and a group of species in the subgenus *Acaudus*) were constrained to state 1 (heteroecious), the likelihood of the reconstruction was significantly worse (more than 2 log units) than when the same nodes were fixed to state 0 (monoecious on herbs) (Table 1). These models and ancestral state assignments comparisons all suggest that, given our dataset, transitions from monoecy on herbs towards heteroecy are likely in this aphid genus. Stochastic mapping suggested similar ancestral character states (Table 2) and a similar total number of life cycle transitions to MP analysis (around 9, Table 3). Like MP, SIMMAP optimisations suggested that heteroecy has evolved from monoecy on herbaceous plants on four or five occasions.

Dietary shifts were also numerous (five transitions estimated by MP and five to seven transitions estimated by stochastic mapping, Table 3, Fig. 4). ML investigation of character state transition suggested that a single rate model was not significantly worse than a two rate model (0.09 log unit difference). We therefore used a single Mk1 model for ML character reconstruction as implemented in Mesquite [28]. The ancestral state in the genus was probably a specialist diet (under all optimisation criteria). MP and ML optimisations suggested several acquisitions of a generalist feeding diet on the secondary host-plant range: one in the subgenus *Brachycaudus *and, potentially, several in the subgenus *Acaudus*, although MP optimisations classified most nodes as equivocal in the subgenus *Acaudus*, indicating that acquisitions and losses were equally parsimonious. SIMMAP results suggested that transitions from a specialist feeding diet to a more generalist feeding diet were more numerous than those in the opposite direction (Table 3).

All monoecious species (except for *B. bicolor*) are specialists, but the posterior predictive test for association between life cycle type and breadth of host range yielded non-significant results (D = 0.19, *p *= 0.11). The lack of statistical association was probably due to the occurrence of several heteroecious species that remained specialised on their secondary host plants.

### Evolution of host-plant association

**Figure 5:**
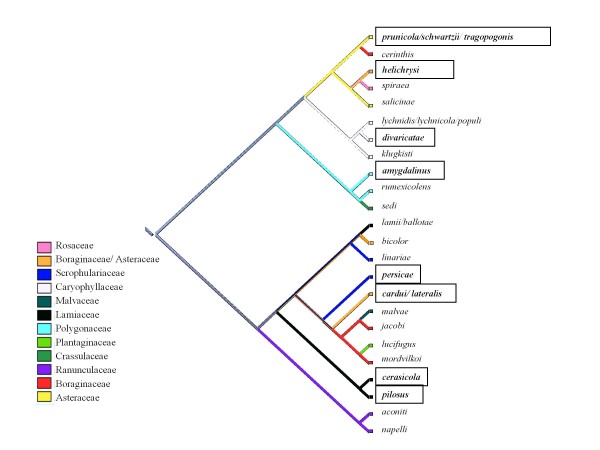
**Evolution of host-plant family associations in the genus *Brachycaudus*. **Framed species are heteroecious.

The mapping of host association on the phylogenetic tree revealed that several monoecious species lived on plants from the same family as the secondary hosts of closely related heteroecious species (Fig. 5). This was the case in the subgenus *Thuleaphis*, in which *B. amygdalinus *(Schouteden, 1905), which alternates between *Prunus amygdalinus *(almond) and *Polygonum *spp. (Polygonaceae), is closely related to *B. rumexicolens*, which is monoecious on plants of the family Polygonaceae (*Rumex spp.*). Similarly, in the subgenus *Brachycaudus*, many of the secondary hosts of heteroecious species and the host plants of monoecious species were found to belong to either the Boraginaceae or Asteraceae. In *Acaudus *species associated with Caryophyllaceae, the only heteroecious species (*B. divaricatae*) alternates between a *Prunus *species and various species of *Silene*, which also act as hosts for monoecious species of this subgenus.

## Discussion

### Evolution of host alternation in Brachycaudus

Our study generated a well resolved phylogeny of the aphid genus *Brachycaudus*. Character mapping revealed the occurrence of multiple transitions in life cycle in this genus during the course of its evolution. Our findings provide strong evidence for the potential evolutionary lability of complex life history traits, such as host alternation. Against all expectations, MP and stochastic mapping both suggested that monoecy on herbaceous host could be the ancestral state in the genus and that up to four (re)-acquisitions of the heteroecious life cycle have occurred. ML optimizations yielded more ambiguities on ancestral character states, and did not allow a precise estimation of the number and types of transitions, but they all favoured a scenario where losses of monoecy on herbs and transitions towards heteroecy have occurred. Character mapping methods are based on prior distributions and models of evolution, the reliability of which remains unclear [29]. But the fact that all optimisation criteria agree on reconstructions involving several reversions towards heteroecy suggests that we can have a high level of confidence in this scenario. Artefacts such as the omission of some species or the existence of asymmetrical diversification rates between monoecious and heteroecious species could bias our results [30,31]. However, available data on described species that were missing from our reconstructions (Additional file 1) indicate that most of them are probably monoecious on herbs; hence their addition should not change our conclusions. Several studies suggest that when a character change has en effect on speciation, inferring transition rates for this character can lead to erroneous conclusions [30]. In our case, monoecy on herbs has been hypothesized to accelerate diversification rates in aphids [32]. This could lead to a high abundance of species that are monoecious on herbs and bias estimation of life cycle transition rates. But this should bias the results towards an overestimation of the number of transitions towards monoecy on herbs. Hence, taking into account biases such as missing species or high diversification of monoecious species on herbs should only confirm that heteroecy can be acquired de novo. Concerning the root of the tree, it is less certain that the actual ancestral state in the genus is monoecy on herbs. *Brachycaudus *belongs to the tribe Macrosiphini, and its closest relatives are often associated with hosts from the Rosaceae (e.g. *Dysaphis *spp. are mostly associated with the genera *Pyrus *and *Malus, Myzus *spp. are often associated with *Prunus *etc.) [15]. Many Aphidinae clearly have a close affinity with rosaceous hosts, and this affinity may be ancestral (as main hosts or primary host for heteroecious species) in some of the tribes of this subfamily [15]. It is therefore likely that some species that have gone extinct over the history of the genus *Brachycaudus *might have been monoecious on a woody host (a *Prunus *or another Rosaceous). We are thus not suggesting that species of the genus *Brachycaudus *have captured, in an independent manner on several occasions, *Prunus*; our study simply demonstrates that potential ancestral rosaceous hosts can easily be recaptured once lost, as the sole host or as primary host for the heteroecious life cycle.

Our results thus clearly contradict the "fundatrix specialisation" hypothesis, which predicts that, once lost, the primary host cannot be recaptured. Moran, in her paper supporting the "fundatrix specialisation" hypothesis [19], rightly pointed out that heteroecious species include some species for which there are differences between populations in the use of secondary herbaceous hosts, with some individuals remaining on primary woody hosts and others alternating between two hosts. By contrast, there have been no reports of variation in the use of the primary host. The absence of "facultative use of the primary host" constitutes a strong argument in favour of the fundatrix specialisation hypothesis, suggesting that host alternation loss involves a loss of the primary host, but not of the secondary host. However, this observation may be biased. As soon as sexual morphs of an aphid species are found on herbaceous hosts, it is generally assumed that the aphid species concerned is monoecious on this host plant and genetically differentiated from any morphologically similar aphids reproducing on a woody host. The subgenus *Appelia *in the genus *Brachycaudus *has been the subject of many taxonomic discussions and revisions [33]. It includes aphids identified as *B. tragopogonis *that are supposedly monoecious on *Tragopogon *spp., aphids identified as *B. schwartzi *that are supposedly monoecious on *Prunus *spp., and aphids identified as *B. prunicola *that are reported as sometimes alternating between various *Prunus *species and *Tragopogon *spp. or monecious on various *Prunus *species (Additional file 1). Even with the use of several variable DNA markers (*Buchnera *intergenic regions), it was not possible to differentiate specimens into these species: this complex of taxa ("subspecies" or "biotypes") may therefore constitute a case of a heteroecious species making facultative use of the primary host for the sexual phase. This may be the "evolutionary step" reported to be missing in Moran's study.

Considering that aphids are the only phytophagous insects exhibiting host alternation, the lability of this trait can be surprising. Furthermore, transitions towards heteroecy necessitate changes in the ability to produce certain morphs (heteroecious species must necessarily produce winged males, while some of the monoecious *Brachycaudus *produced unwinged males only [14]) as well as changes in host preference for *Prunus *in several morphs (winged males and gynoparae). This a priori involves complex changes that are not likely to occur repeatedly during the course of evolution. However aphid clones have the ability to produce alternative morphs which is also very unusual among phytophagous insects. Further, the developmental processes underlying morph determination seem quite labile as for instance, the production of winged and unwinged males is highly variable across the phylogeny [34] and some species in the *Brachycaudus *genus and many other aphids genera produce both types of males [14]. Transcriptomic analyses actually suggest that alternative morphologies can be determined by alternative gene expression [34]. This ability to produce alternative morphs with the same genetic material might actually facilitate evolutionary changes in life cycles [35]. Concerning the use of rosaceous hosts in *Brachycaudus*, its lability is actually not particularly surprising. Retention of the ability to use former host plants may be quite common in phytophagous insects [36]. Transitions towards heteroecy simply represent another example of reacquisition of ancestral character states [37,38]. Finally, phylogenetic studies of life cycle evolution in parasites have suggested that complex life cycles are evolutionary labile [39-41], and evolutionary research in the domain of parasitology has largely focused on the selective advantage of using several hosts rather than on the constraints limiting this trait [42,43]. Hence the lability of life cycle in aphids is not that surprising when put into perspectives with literature on life cycle evolution in other groups.

The alternative hypothesis to the "fundatrix specialisation hypothesis" is that heteroecy is advantageous. However, this raises a question already posed in a previous study [16]: why would closely related species living in similar environments have different life cycles? Furthermore, the presence of fewer host-alternating species in the genus than of monoecious species, and the small number of heteroecious aphids in general (only 10% of all aphid species) are not consistent with the hypothesis that host alternation is selectively advantageous, unless this advantage applies to only a very small number of environments. We suggest that the observed patterns of life cycle evolution in the genus *Brachycaudus*, and possibly similar patterns in other genera of the Aphidinae, probably result from a combination of both selection and constraints on host plant choices. Studies of closely related species with different life cycles should improve our understanding of the balance between these forces. These biological models could be used to study morphological changes in the fundatrix following the evolutionary loss of the primary host. Indeed, such an approach would actually be the best way to test the fundatrix specialisation hypothesis [19]. The genetic determinants of aphid morphs involved in life cycle variation (i.e. winged, sexual morphs) have also been little studied, and may actually involve very few genes [44] or genes that are easily switched on and off [31], making the reacquisition of a woody host less unlikely than previously thought. This could be investigated with experimental data in the genus *Brachycaudus*, but the genomic information now available for aphids could also be used [45]. Such studies are necessary to give definitive proofs of the lability of life cycle in the genus *Brachycaudus*.

### Consequences of host alternation for host-plant association and the diversification of aphids

Host-plant associations are highly diversified in the genus *Brachycaudus*. Our phylogenetic tree revealed two patterns of host-plant evolution: 1) several monoecious species were found to be associated with herbaceous host plants from the same family as the secondary hosts of closely related heteroecious species; 2) many monoecious species were found to be associated with host plants unrelated to the heteroecious life cycle. For example, *Brachycaudus malvae*, which is associated with *Malva *spp., is closely related to heteroecious and monoecious species associated with plants of the Asteraceae.

The first of these patterns, when found in other genera of Aphidinae, has been interpreted as an illustration of speciation via loss of the heteroecious life cycle and the shifting of all primary host generations onto some of the secondary hosts of the original life cycle [13]. This scenario has been suggested for some species in the genus *Cryptomyzus *[46]. For example, *C. alboapicalis *is monoecious on mints (Lamiaceae), whereas closely related species, such as *C. galeopsidis*, alternate between a woody host (*Ribes *spp.) and mint [47]. *C. alboapicalis *is thought to have differentiated from its sister species by losing the primary woody host. Our study in the genus *Brachycaudus *suggests an additional diversification scenario, in which re-acquisitions of heteroecy have probably played a major role in speciation events. A shift in life cycle, so long as it involves the loss or acquisition of the primary (winter) host in a group of individuals of an aphid species, necessarily results in the shifting of sexual reproduction to a separate host plant and strong reproductive isolation from the population of origin. Hence, the multiple acquisitions of heteroecy revealed by our study have probably promoted the diversification of some *Brachycaudus *species. Aphidinae, the aphid subfamily with the largest number of species, includes many genera displaying host alternation. Some genera, like the genus *Brachycaudus*, include both monoecious and heteroecious species (e.g. *Dysaphis*, *Cryptomyzus*, *Cavariella*, *Metopolophium*, *Capitophorus*). It would be interesting to carry out phylogenetic investigations in these genera to determine whether the acquisition or re-acquisition of heteroecy has also occurred and could account for some of the diversification events.

The second pattern, the use of host plants unrelated to the heteroecious life cycle, is more difficult to explain. How did aphids capture these new hosts? We found no significant correlation between transitions in diet and changes in life cycle, but the secondary host-plant repertoire of heteroecious species is often larger than the host-plant repertoire of monoecious species. This larger host plant repertoire on secondary host plants is a common pattern in aphids [7], and may favour the capture of new hosts [9,15]. The genus *Brachycaudus *may illustrate speciation via the acquisition of new, sometimes distantly related hosts during the "summer" phase of the heteroecious life cycle.

## Conclusions

In conclusion, our results suggest that host alternation is surprisingly labile in the aphid genus *Brachycaudus*. The primary woody hosts can be lost and easily recaptured. These results clearly go against "the fundatrix specialisation" hypothesis, suggesting that aphids in the genus *Brachycaudus *and, possibly, other genera of the Macrosiphini tribe, seem to have escaped from the hypothetical dead end put forward by Moran [7]. Improvements in our understanding of the selective forces behind the evolution of host alternation in aphids will require elucidation of the genetic determinants of the fundatrix and the sexual morphs and of the oviposition preferences of sexual morphs and thorough ecological studies of closely related species with different life cycles or natural populations of species described as facultatively heteroecious. There are several good candidate species in the genus *Brachycaudus *for comparative analyses of this type.

## Methods

### Sampling and phylogenetic reconstruction

The taxa used here were those used in a previous study [23], with the addition of three species: *Brachycaudus sedi*, *Brachycaudus cerasicola *and *Brachycaudus pilosus*, the only representative of the subgenus *Mordvilkomemor *(sensu [2]). We also added several specimens from various species used in our previous reconstructions. All specimens were identified by AC. We studied 88 specimens from 30 of the 43 recognised species in the genus and three specimens identified as *B*. (*Thuleaphis*) sp. obtained from Kazakhstan (Central Asia) that we were unable to identify, but which may belong to a new species described by Kadyrbekov [26,27] (collection details are given in Additional file 2).

Sequences were obtained for these new taxa by the same methods used in previous studies [23,24]. Six DNA fragments were amplified and sequenced: two mitochondrial DNA regions (COI and CytB), one aphid nuclear DNA region (ITS), three *Buchnera aphidicola *DNA fragments (Trpb and two intergenic regions, sbf-dna and hupa-rpoc). A second nuclear DNA marker was also added to complete the dataset, as the ITS fragment was poorly informative beyond the subgenus level. We used primers developed in a previous study [48] on *Aphis gossypii *Glover 1877 to amplify and sequence a portion of an intergenic region in the sodium channel para-type gene (hereafter referred to as the Aph marker). We have previously shown that: 1) aphid nuclear DNA (ITS) and mitochondrial DNA phylogenetic results are consistent [24]; 2) there is perfect cospeciation between *Buchnera aphidicola *associated with *Brachycaudus *and their hosts, validating the use of *Buchnera *DNA for reconstructing the phylogenetic relationships of aphid species in the genus *Brachycaudus *[23]. We therefore analysed a combined DNA dataset, corresponding to 4734 aligned bp after the exclusion of two ambiguously aligned zones of about 200 bp each in *Buchnera *intergenic fragments. All sequences have been submitted to GenBank (accession numbers GU568382-GU568672). Coding regions (the Cytb, COI, and *Buchnera *Trpb genes) were checked for frameshifts in the coding frame with Mega 3 software [49].

Phylogenetic analyses were based on maximum likelihood (ML) analyses and Bayesian inference.

For ML reconstructions, the model of nucleotide substitution was selected in Model Test 3.7 [50]. The MP tree with the highest Ln score was used to estimate the model parameters (gamma shape, base frequencies, substitution matrix). A ML heuristic search, using a starting tree obtained by MP methods, was then conducted in PhyML [51], using the selected model. Node support was assessed with the bootstrap technique, with 500 replicates.

Bayesian phylogenetic analyses were conducted in MrBayes 3.1.2 [52]. Sequence data were partitioned by gene region, this partitioning strategy having been identified as the best fitting model with Bayes factors [23]. We used the GTR + I+ G model identified as the best-fit model for all DNA fragments. The parameters of the model were treated as unknown variables with uniform prior probabilities and were estimated during the analysis. They were allowed to vary across partitions. Two replicate analyses were run for 5 million generations. For each replicate, we ran one cold chain and three hot chains of the Markov Chain Monte Carlo method, using a random starting tree and sampling trees every 100 generations. The point of stationarity was determined as the point at which the distribution of likelihoods reached a plateau and trees prior to stationarity were discarded (5000 trees). The remaining trees were used to calculate 50% majority rule consensus trees. Posterior probabilities (pp) were summarized accordingly.

### Species phylogeny

We applied the method developed by Pons *et al*. [53], which identifies genetic clusters representing independently evolving entities using a generalised mixed Yule model. This method statistically differentiates the shift in the branching patterns of a phylogenetic tree, from interspecific long branches to intraspecific short polytomous branches, using likelihood approaches [53]. It first checks that the specimens do not all belong to a single population obeying a single coalescent process. Under this assumption, an optimal threshold is identified such that nodes before this threshold are considered as species diversification events, whereas branches crossing the threshold define clusters following a coalescent process.

The GMYC model was run on an ultrametric tree generated by Multidivtime analysis. The Multidivtime analysis was run as previously explained [23], adding a new partition to the dataset (Aph partition), together with sequences for the new specimens. As described in the manual [54], the parameters of the substitution model used by Estbranches were estimated, for each partition separately, with the baseml program of the PAML package [55]. The output from baseml was then used for the first step of the Multidistribute package: paml2modelinf was run to convert these outputs into data useable by Estbranches. This program produces ML estimates of branch lengths within the optimal tree topology estimated from the combined data (we used the ML tree topology) and a variance-covariance matrix for each locus. Multidivtime then makes use of these output files to estimate divergence times. We used the default setting for Markov chain Monte Carlo analyses (100,000 cycles in which the Markov chain was sampled 10,000 times every 100^th ^cycle after burn in). Ultrametric trees for species delimitation analysis were obtained by arbitrarily assigning prior ages of 1.0 (SD = 1). Rtrate (mean rate of molecular evolution at the ingroup root node) was estimated by calculating the median branch length from the root to ingroup tips.

### Reconstruction of character evolution

Our Bayesian consensus tree was well resolved and well supported, but we nonetheless accounted for phylogenetic uncertainty and branch length variation in character reconstruction by randomly selecting 100 trees from the stabilised part of the Markov Chain Monte Carlo analysis. For this set of trees, we derived a set of "phylogenetic species" trees based on the results of the species delimitation method, by picking (at random) one specimen for each putative species and pruning subsequent specimens from the global tree with TreeEdit [56]. We obtained 100 different "phylogenetic species phylograms", on which we mapped life cycle evolution and host range breadth using several methods of optimization.

We treated "life cycle" as a three-state character: monoecious on herbaceous hosts (0), heteroecious (1), monoecious on woody hosts (2). We used the data on life cycle and host plant association summarised in previous studies [22,57], using additional references to resolve ambiguities (see Additional file 1 for a summary of ecological data for each species in the genus). When species were identified as facultatively heteroecious (some populations do not alternate on different hosts, or there may have been confusion in the literature when identifying these species, see Additional file 1), we conducted two analyses. In the first analysis, these species were treated as heteroecious, whereas in the second, they were treated as monoecious (on woody hosts). When monoecious species and heteroecious species where lumped together in a single genetic cluster by the species delimitation method, this "cluster" was coded as "heteroecious".

Host range breadth is a continuous character that is difficult to define. Heteroecious *Brachycaudus *species may be specialists on their primary host plants but generalists on their secondary host plants. In this analysis, for heteroecious species, we consider this feature only for secondary hosts. Specialists (0) were defined as species feeding on a single plant species or a few plant species belonging to one or two genera. Generalists (1) were defined as species feeding on several host plants, from more than two genera, even belonging to more than two families, in some cases. When "taxonomic" species were lumped together, the breadth of the host range of the resulting "phylogenetic species" was re-estimated, taking into account the host plant repertoire of all the species clustered together.

Many different optimisation criteria and models are used for reconstruction of the ancestral state of phenotypic characters, resulting in potentially very different conclusions [29]. We thus used several methods and compared their results. Character reconstructions were first achieved by maximum parsimony (MP) methods, as implemented in Mesquite version 2.7.1 [28]. Maximum likelihood (ML) reconstructions were also conducted using the program BayesMultistate [58] incorporated in the computer package BayesTraits. This method allows testing hypotheses about transition rates and ancestral character states using Likelihood Ratio tests. We first tested the fit of several models of evolution for both characters. For life cycle, we first fitted a full model with six parameters representing all possible changes between the three character states and then a simplified model with a single rate for all transitions. As our aim was to test some of the predictions of the fundatrix specialisation hypothesis, we also fitted a model setting secondary gains of a woody host (i.e. transition rates from monoecy on herbs towards heteroecy and monoecy on woods) to 0. We compared this model to a model where reverse transitions were set to 0. When comparing models, as they were generally not nested, we applied the general rule of thumb that two log likelihood units constitutes a significant difference [3,59]. We then reconstructed ancestral states for 12 nodes of interest, all of which were supported by pp values > 95%. Still aiming at testing hypotheses concerning life cycle evolution, we compared the likelihood of the reconstructions when several internal nodes were fixed to state (0) monoecious on herbs (using the "fossil node" command in Multistate) to the likelihood of the reconstructions when these same nodes were fixed to state (1) heteroecious. For feeding diet evolution, we compared the fit of a two rate model (asymmetric model) to the fit of a single rate model (mk1) model and using the preferred model, we reconstructed ancestral states for several nodes of interest. Finally the Bayesian stochastic mapping approach [60], implemented in the software SIMMAP v.1.0 [61], was also used. In this analysis, we estimated the posterior probabilities of character states for 12 nodes. Random determinations of character histories consistent with the character states observed at the tips of the tree were used to estimate posterior probabilities for specific ancestral state reconstruction. SIMMAP can use a symmetric beta prior distribution (bias parameter) for the morphological state frequencies of binary characters and a prior distribution for the overall transformation rate for all characters. We explored the effect of different prior distributions on character reconstruction. For diet (a binary character), we set the α parameter, describing the shape of the distribution, at 1, resulting in a flat prior distribution, i.e. the transition rates from one state to another are not a priori considered as equal (the distribution was discretised into 19 categories). For the overall rate of evolution for both characters, we first tried a high rate of evolution, assuming that characters could evolve rapidly: the mean prior tree length E(T) was set to 10 and the SD to 1, by setting α = 100 and β = 10. We then tried a lower prior rate of evolution, with the mean prior tree length E(t) set to 1.5 by setting α = 3 and β = 2 (the gamma distribution was discretised into 50 categories). For each character reconstruction, for each of the 100 trees, 50 stochastic draws from the prior distributions of characters rates of evolution were taken. Total tree lengths were rescaled to one before including the morphology priors. We obtained data for the number of transformations and transition rates for each character, for each set of priors, using the "simulate history" option in SIMMAP. For these simulations, we used 10 runs from the priors and 10 runs for each tree and each character.

We assessed the association between life cycle state and host range, using the posterior predictive test implemented in SIMMAP. The association test statistic, D, measures the difference between expected and observed frequencies of character states for two characters occurring together on a tree. We used the same set of priors as above, with 10 runs from the priors and 10 runs for each tree and each character.

### Evolution of host-plant association

The objective here was not to reconstruct ancestral states, but simply to find a convenient way to visualise the evolution of host-plant association. In particular, we wanted to determine whether monoecious species used plants from the secondary host-plant range of closely related heteroecious species. We therefore characterised host-plant family for each species and mapped the host-plant family of monoecious species and the secondary host-plant family of heteroecious species. The genus includes two highly polyphagous species, *Brachycaudus cardui *and *Brachycaudus helichrysi*, both of which associated mostly with Asteraceae and Boraginaceae. We therefore assigned these species a character state for host association, lumping these two plant families together. The host-plant family association of *Brachycaudus *species had 13 states (i.e. *Brachycaudus *species were associated with 13 different plant families). We mapped the evolution of host-plant association, using the parsimony ancestral state reconstruction tool of Mesquite on the chronogram obtained with Multidivtime.

Species for which no biological information had been published (undescribed species) were discarded from the analyses (the corresponding taxa were pruned from the tree). Outgroup taxa were excluded from the ancestral character reconstructions.

## Authors' contributions

EJ and AC co-designed the study and conducted specimen sampling. EJ participated in sequence acquisition and alignment, conducted phylogenetic analyses and drafted the MS. GG participated in DNA sequence acquisition and analyses. AC conducted all taxonomic identifications, and participated in the writing of the paper. All authors read and approved the final manuscript.

## Supplementary Material

Additional file 1 **Life cycle and host plant associations of *Brachycaudus *species**. Click here for file

Additional file 2 **Sample information**. Click here for file
