# Supplementary material for: Evolutionary lability of a complex life cycle in the aphid genus Brachycaudus
Source: BMC Evol Biol. 2010 Sep 28;10:295. doi: 10.1186/1471-2148-10-295 (PMC2958166; doi:10.1186/1471-2148-10-295)
Supplement: Additional file 1 — Life cycle and host plant associations of Brachycaudus species. [file 1471-2148-10-295-S1.DOC]

Table S1: Life cycle and host plant associations of *Brachycaudus* species from [22, 57]. Species included in our phylogenetic reconstructions are in bold character and are preceded with a *.

|  | Life cycle | Host association |
| --- | --- | --- |
| *Subgenus Brachycaudus* van der Goot, 1913 | | |
| *B. divaricatellus* Shaposhnikov, 1956 | Life cycle unknown, monoecious on ligneous or heteroecious | *Prunus divaricata* Ledeb.(Rosaceae) |
| ****B. helichrysi* (Kaltenbach, 1843)** | Heteroecious with some anholocyclic populations | Primary host: *Prunus* spp. (Rosaceae), polyphagous on secondary hosts, mostly Asteraceae and Boraginaceae |
| ****B. salicinae* Börner, 1939** | Monoecious on herbs | *Inula* spp. especially *Inula salicina* L. (Asteraceae) |
| *B. shaposhnikovi* Narzikulov, 1949 | Monoecious on ligneous | *Atraphaxis pyrifolia* Bunge (Polygonaceae) |
| ****B. spiraeae* Börner, 1932** | Monoecious on ligneous | *Spiraea* spp. (Rosaceae) |
| *Subgenus Acaudus* van der Goot, 1913 | | |
| ****B. aconiti* (Mordvilko, 1928)** | Monoecious on herbs | *Aconitum* spp. and *Delphinium* spp. (Ranunculaceae) |
| *B. almatinus* (Nevsky, 1951) | Life cycle unknown, monoecious on ligneous or heteroecious | *Prunus* sp. *Pyrus* sp*.* (Rosaceae) |
| ****B. ballotae* (Passerini, 1860)** | Monoecious on herbs | *Ballota nigra* L. (Lamiaceae) |
| ****B. cardui* (Linnaeus, 1758)** | Heteroecious | Primary host: *Prunus* spp. mostly *P. domestica* L. (Rosaceae), main secondary hosts in Asteraceae and Boraginaceae |
| ****B. cerasicola* (Mordvilko, 1929)** | Heteroecious | Primary host: various *Prunus* spp. (Rosaceae), secondary hosts: various species of Lamiaceae |
| ****B. divaricatae* Shaposhnikov, 1956** | Monoecious on ligneous [62] or heteroecious [63] | Primary host or sole host: *Prunus* sp. (Rosaceae), with facultative alternation on *Silene* spp. (Caryophyllaceae) |
| *B. gentianae* Daniyarova, 1990 | Probably monoecious on herbs [sexual morphs unrecorded] | *Gentiana olgae* Rgl. et Schmalh. (Gentianaceae) |
| *B. iranicus* Davatchi & Remaudière, 1953 | Monoecious on herbs | *Anchusa azurea* Mill.*, Anchusa strigosa* Labill. (Boraginaceae) |
| ****B. jacobi* Stroyan, 1957** | Monoecious on herbs | *Myosotis* and *Pulmonaria* spp. (Boraginaceae) |
| ****B. klugkisti* (Börner, 1942)** | Monoecious on herbs | Reported on various *Silene* spp. (Caryophyllaceae), especially *S. dioica* (L.) Clairv. and *S. latifolia* (L.) Clairv. [14] |
| ****B. lamii* (Koch, 1854)** | Monoecious on herbs | *Lamium* spp. especially *L. album* L. (Lamiaceae) |
| ****B. lateralis* (Walker, 1848)** | Mainly anholocyclic but occasionally heteroecious (with a sexual phase on *Prunus* sp.) | Polyphagous with main hosts in Asteraceae |
| ****B. linariae* Stroyan, 1950** | Probably monoecious on herbs ^a^ [sexual morphs unrecorded] | *Linaria* spp. (Scrophulariaceae) |
| ****B. lucifugus* F.P. Müller, 1952** | Monoecious on herbs | *Plantago lanceolata* L. (Plantaginaceae) |
| ****B. lychnicola* Hille Ris Lambers, 1966** | Monoecious on herbs | Reported on various *Silene* spp., especially *S. flos-cuculi* L. and *S. dioica* (L.) Clairv. (Caryophyllaceae)[14] |
| ****B. lychnidis* (Linnaeus, 1758)** | Monoecious on herbs | Reported on various *Silene* spp., especially *S. dioica* (L.) Clairv. and *S. latifolia* Poiret (Caryophyllaceae)[14] |
| ****B. malvae* Shaposhnikov, 1964** | Probably Monoecious on herbs [sexual morphs unrecorded] | *Malva* spp. (Malvaceae) |
| ****B. mordvilkoi* Hille Ris Lambers, 1931** | Monoecious on herbs | Several Boraginaceae (*Anchusa*, *Echium*) |
| ****B. napelli* (Schrank 1801)** | Monoecious on herbs | *Aconitum* spp. (especially *A.* *napellus* L.) and occasionally *Delphinium* spp. (Ranunculaceae). |
| *B. pallidus* Andreev, 1990 | Probably monoecious on herbs [sexual morphs unrecorded] | *Silene nutans* L. (Caryophyllaceae) |
| ****B. persicae* (Passerini 1860)** | Monoecious on ligneous or heteroecious, depending on the study | Primary host or sole host: *Prunus* spp.(Rosaceae) especially *P*. *spinosa* L. with facultative alternation on various Scrophulariaceae [14] |
| *B. plantaginis* Holman & Szelegiewicz, 1975 | Probably monoecious on herbs [sexual morphs unrecorded] | *Plantago depressa* Willd., *P. major* L. (Plantaginaceae)r |
| ****B. populi* (del Guercio, 1911)** | Monoecious on herbs | Reported on various *Silene* spp., especially *S. vulgaris (Moench.) Garcke* (Caryophyllaceae) [14] |
| *B. rinariatus* Andreev, 1982 | Probably monoecious on herbs [sexual morphs unrecorded] | *Linaria vulgaris* Miller (Scrophulariaceae) |
| *B. rociadae* (T.D.A. Cockerell, 1903) | Monoecious on herbs | *Delphinium* spp. (Ranunculaceae) |
| *B. virgatus* Shaposhnikov, 1964 | Probably monoecious on herbs [sexual morphs unrecorded] | *Anchusa* sp. *Symphytum* sp. (Boraginaceae), *Senecio* *fluviatilis* Wallr. (Asteraceae) |
| *Subgenus Appelia* Börner, 1930 | | |
| ****B. cerinthis* Bozhko, 1961** | Monoecious on herbs | *Cerinthis* spp. (Boraginaceae) |
| ****B. prunicola* (Kaltenbach, 1843)** | Facultatively heteroecious | Primary host or sole host: *Prunus* spp. (Rosaceae), potential secondary hosts: *Tragopogon* spp. (Asteraceae) |
| ****B. schwartzi* (Börner, 1931)** | Monoecious on ligneous | *Prunus* *persicae (L.) Batsch* (Rosaceae) |
| ****B. tragopogonis* (Kaltenbach, 1843)** | Monoecious on herbs | *Tragopogon* spp. (Asteraceae) |
| *Subgenus Mordvilkomenor* Shaposhnikov, 1950 | | |
| ****B. pilosus* (Mordvilko, 1929)** | Monoecious on ligneous or heteroecious, depending on the study (this species has been often confused with *B. cerasicola*) | Primary host or sole host: *Prunus* spp.(Rosaceae), with facultative alternation on secondary hosts of the Lamiaceae family |
| *Subgenus Nevskyaphis* Shaposhnikov, 1950 | | |
| ****B. bicolor* (Nevsky, 1929)** | Probably monoecious on herbs ^a^ [sexual morphs unrecorded] | Various species in several genera of Boraginaceae, also reported on a few species of Asteraceae |
| *Subgenus Thuleaphis* Hille Ris Lambers, 1960 | | |
| *B. acaudatus* (Hille Ris Lambers, 1960) | Monoecious on herbs | *Persicaria viviparum* (L.) Ronse Decr. |
| ****B. amygdalinus* (Schouteden, 1905)** | Heteroecious and sometimes anholocyclic on Polygonaceae | Primary host: *Prunus* *amygdalus* Batsch (Rosaceae)., secondary hosts: *Polygonum* spp., *Persicaria maculosa* S.F. Gray, *Fagopyrum cymosum* (Trev.) Meisn (Polygonaceae) |
| *B. brevirostratus* Pashtshenko, 1988 | Probably monoecious on herbs (possibly a synonym of *B. rumexicolens* [48]) | *Polygonum* spp. (Polygonaceae) |
| *B. eurotiae* (Mamontova, 1968) | Probably monoecious on ligneous | *Krascheninnikovia ceratoides* (L.) Gueldenst. (Chenopodiaceae) |
| ****B. rumexicolens* (Patch, 1917)** | Monoecious on herbs | *Rumex acetosella* L. and sometimes other Polygonaceae (*Fagopyrum*, *Polygonum*) |
| ****B. sedi* (Jacob, 1964)** | Monoecious on herbs | *Rhodiola rosea* L. (Crassulaceae) |
| ****B.* sp.** | Unknown | *Athraphaxis* spp. (Polygonaceae)- ligneous host |

^a^ Though the life cycle of these species is unknown, as sexuals have not been found, it seems unlikely that they are heteroecious, or at least that they use *Prunus* as a host during their life cycle as we have sampled extensively on many *Prunus* species in the distribution range of these species in France and Kazakhstan during the course of this study and have not found them on any *Prunus*.
