# Supplementary material for: Evolutionary lability of a complex life cycle in the aphid genus Brachycaudus
Source: BMC Evol Biol. 2010 Sep 28;10:295. doi: 10.1186/1471-2148-10-295 (PMC2958166; doi:10.1186/1471-2148-10-295)
Supplement: Additional file 2 — Sample information. [file 1471-2148-10-295-S2.DOC]

Table S2 : Sample information

| Species | Voucher | Collectors | Collection site | Host plant |
| --- | --- | --- | --- | --- |
| *B. aconiti* | 1790 | Coeur.& Jous. | France, Ariège (09), Mijanes, Col de Pailhères | *Aconitum sp.* |
| *B. amygdalinus* | 1688 | Coeur.& Jous. | France, Var (83), Fayence | *Prunus dulcis* |
|  | **1689** | **Coeur.& Jous.** | **France, Var (83), Fayence** | ***Prunus dulcis*** |
|  | 1694 | Coeur.& Jous. | France, Bouches-du-Rhône (13), St-Martin-de-Crau | *Prunus dulcis* |
|  | 1710 | Coeur.& Jous. | France, Gers (32), Saint-Clar | *Prunus dulcis* |
|  | **2538** | **Coeur.& Jous** | **Kazakhstan, Ongtustik, Zhabagly** | ***Polygonum* *aviculare*** |
| *B. ballotae* | s338 | G. Cocuzza | Germany, Berlin | *Ballota nigra* |
| *B. bicolor* | 1458 | Coeur d'Acier | Greece, Lakonia, Lagada | Boraginaceae sp. |
|  | **1328** | **Coeur d'Acier** | **France, Morbihan (56), Saint-Pierre-Quiberon** | unknown |
|  | **2521** | **Coeur.& Jous** | **Kazakhstan, Almaty, Medeu** | *Echium sp.* |
| *B. cardui* | 1709 | Coeur.& Jous. | France, Haute-Garrone (31), Grenade | Asteraceae sp*.* |
|  | 1746 | Coeur.& Jous. | France, Haut-Rhin (68), Colmar | *Prunus domestica* |
|  | 1765 | Coeur.& Jous. | France, Gard (30), Le Vigan | *Arctium sp.* |
|  | **2215** | **Coeur.& Jous.** | **Kazakhstan, Ongtustik, Zhabagly** | ***Prunus sp****.* |
|  | **2549** | **Coeur.& Jous.** | **Kazakhstan, Ongtustik, Zhabagly** | *Carduus sp.* |
| *B. cerinthis* | 1772 | Coeur.& Jous. | France, Hautes-Alpes (05), Villar-d'Arene | *Cerinthe glabra* |
| ***B. cerasicola*** | **2554** | **Coeur.& Jous.** | **Kazakhstan, Ongtustik, Zhabagly** | | ***Betonica foliosa*** | | --- | |
| *B. divaricatae* | s242 | G. Cocuzza | Lithuania, Vilnius, Bratoskies | *Prunus divaricata* |
| *B. helichrysi* | 1681 | Coeur.& Jous. | France, Bouches-du-Rhône (13), St Martin de Crau | *Senecio sp.* |
|  | 1695 | Coeur.& Jous. | France, gard (30), Caveirac | *Prunus domestica* |
|  | **1712** | **Coeur.& Jous** | **France, Gers (32), St Clar** | ***Prunus domestica*** |
|  | **1728** | **Coeur.& Jous** | **France, Hérault (34), Montpellier** | ***Prunus domestica*** |
|  | 1749 | Coeur.& Jous | France, Doubs (25), Pontarlier | *Prunus domestica* |
|  | **1828** | **Coeur.& Jous.** | **Australia, Queensland, Cardwell** | **Asteraceae sp*.*** |
|  | **2170** | **Coeur.& Jous.** | **Kazakhstan, Almaty** | ***Prunus sp.*** |
|  | **2218** | **Coeur.& Jous.** | **Kazakhstan, Ongtustik, Zhabagly** | *Prunus domestica* |
|  | **2590** | **Coeur.& Jous.** | **Kazakhstan, Almaty** | **Asteraceae sp*.*** |
| *B. jacobi* | s145 | G. Cocuzza | Italy, Sicily, Itala | *Myosotis sylvatica* |
| *B. klugkisti* | 1290 | Coeur d'Acier | France, Creuse (23), Peyrat-la-Noniere | *Silene* sp. |
|  | 1747 | Coeur.& Jous. | France, Haut-Rhin (68), S^te^-Marie-Aux-Mines | *Silene dioica* |
|  | 2063 | Jousselin | France, Haute-Savoie (74), La Roche sur Foron | *Silene dioica* |
|  | 2064 | Coeur.& Jous. | France, Pyrénnées Orientales, | *Silene dioica* |
| *B. lamii* | s328 | G. Cocuzza | Italy, Sicily, Montalbano Elicona | *Lamium flexsuosum* |
| *B. lateralis* | 1027 | Coeur d'Acier | France, Finistère (29), Cleden-Cap-Sizun | *Senecio jacobaea* |
|  | 1741 | Coeur.& Jous. | France, Drôme (26), St-Marcel-les-Valence | *Senecio* sp*.* |
|  | 1751 | Coeur.& Jous. | France, Haut-Rhin (68), Colmar | *Arctium* sp*.* |
|  | s117 | G. Cocuzza | Italy, Sicily, Salina | *Chrysanthemum coronarium* |
|  | 1794 | Coeur.& Jous. | France, Lozère (48), La Bastide-Puylaurent | *Senecio* sp*.* |
| *B. linariae* | **1771** | **Coeur.& Jous** | **France, Hérault (34) St-Guilhem-le-Desert** | ***Linaria repens*** |
|  | 1938 | Coeur.& Jous. | France, Hérault (34) Prades le lez, CBGP | *Linaria repens* |
|  | 2047 | Coeur.& Jous | Italy, Sicilia, Zafferana | *Linaria purpurea.* |
| *B. lucifugus* | s249 | G. Cocuzza | Italy, Trentino Alto Adige, Ala | *Plantago lanceolata* |
| *B. lychnicola* | s317 | G. Cocuzza | Czech Republic, South Bohemia, Lužanská Udolí | *Silene flos-cuculi* |
| *B. lychnidis* | 1324 | Coeur d'Acier | France, Morbihan (56), Saint-Pierre-Quiberon | *Silene* sp. |
|  | 1698 | Coeur.& Jous. | France, Hérault (34) St-Guilhem-le-Desert | *Silene latifolia* |
|  | 1752 | Coeur.& Jous. | France, Haut-Rhin (68), Colmar | *Silene latifolia* |
|  | 1762 | Coeur.& Jous. | France, Gard (30), Le Vigan, Col de Faubel | *Silene dioica* |
| *B. malvae* | s125 | G. Cocuzza | Italy, Lazio, Roma | *Malva sylvestris* |
| *B. mordvilkoi* | s248 | G. Cocuzza | Italy, Trentino Alto Adige, Ala | *Echium vulgare* |
| *B. napelli* | s316 | G. Cocuzza | Czech Republic, South Bohemia, Lužanská Udolí | *Aconitum callybotrium* |
| *B. persicae* | 1077 | Coeur d'Acier | France, Aude (11), Quillan, La Forge | *Prunus spinosa* |
|  | 1696 | Coeur.& Jous. | France, | *Prunus* sp*.* |
|  | **1701** | **Coeur.& Jous.** | **France, Gers (32), St Clar** | ***Prunus spinosa*** |
|  | 1736 | Coeur.& Jous. | France, Drôme (26), St-Marcel-les-Valence | *Prunus* sp*.* |
| *B. populi* | 1483 | Coeur d'Acier | Greece, Lakonia, Mystra | *Silene vulgaris* |
|  | 1760 | Coeur.& Jous. | France, Gard (30), Le Vigan, Col de Faubel | *Silene vulgaris* |
| *B. prunicola* | **992** | Coeur d'Acier | **France, Finistère (29), Fouesnant** | ***Prunus spinosa*** |
|  | 1267 | Coeur d'Acier | France, Creuse (23), Vallieres, La Prades | *Prunus* sp*.* |
|  | **1990** | **Coeur.& Jous** | **Sicily, Catania, Castiglione di Sicilia** | ***Prunus spinosa*** |
|  | **2534** | **Coeur.& Jous** | **Kazakhstan, Ongtustik, Zhabagly** | *Prunus* sp*.* |
|  | **2550** | **Coeur.& Jous** | **Kazakhstan, Ongtustik, Zhabagly** | *Prunus* sp*.* |
|  | **2574** | **Coeur.& Jous** | **Kazakhstan, Ongtustik, Kornilovskoyey** | *Prunus* sp*.* |
| *B.rumexicolens* | 1764 | Coeur.& Jous. | France, Gard (30), Le Vigan, Col de Faubel | *Rumex acetosella* |
|  | 1982 | Coeur.& Jous | Italie, Sicile, Linguaglossa | *Rumex acetosella* |
| *B. salicinae* (Börner, 1939) | s307 | G. Cocuzza | Czech Republic, South Bohemia, Českỳ Krumlov | *Inula salicina* |
| ***B. sedi*** | **2154** | **Coeur.& Jous** | **United Kingdom, Scotland, Glencoe** | ***Rhodiola rosea*** |
|  | **2298** | **Coeur.& Jous** | **France, savoie (73), Bessans** | ***Sedum sp.*** |
|  | **2608** | **Coeur.& Jous** | **France, savoie (73), Bessans** | ***Sedum*** ***anacampseros*** |
| *B. schwartzi* | 1717 | Coeur.& Jous. | France, Tarn-et-Garonne (82), Gramont, Hameau de Géran | *Prunus persica* |
|  | 1730 | Jousselin | France, Centre, Loiret (45), Germigny-Des-Pres | *Prunus persica* |
|  | 1775 | Coeur.& Jous. | France, Hautes-Alpes (05), La Grave | *Spiraea* sp. |
| *B.spiraeae* | 2143 | Coeur.& Jous. | United Kingdom, Scotland, Kinlochewe, | *Spiraea salicifoliae* |
|  | **2226** | **Coeur.& Jous** | **Kazakhstan, Ongtustik, Zhabagly** | ***Spiraea sp*** |
|  | **2240** | **Coeur.& Jous** | **Kazakhstan, Almaty, Talghar** | ***Spiraea sp.*** |
|  | **2586** | **Coeur.& Jous** | **Kazakhstan, Ongtustik, Zhabagly** | ***Spiraea hypericifolia*** |
| *B. tragopogonis* | 1378 | Coeur d'Acier | Greece, Korinthia, Némea | *Tragopogon sp.* |
|  | 1715 | Coeur.& Jous. | France, Tarn-et-Garonne (82), Gramont, Hameau de Géran | *Tragopogon sp.* |
|  | **1750** | **Coeur.& Jous.** | **France, Haut-Rhin (68), Colmar** | ***Tragopogon sp*** |
|  | 1773 | Coeur.& Jous. | France, Hautes-Alpes (05), Villar-d’Arene | *Tragopogon sp.* |
|  | **2522** | **Coeur.& Jous** | **Kazakhstan, Almaty, Medeu** | ***Tragopogon sp*** |
| ***B. sp 1 (Thuleaphis)*** | **2195** | **Coeur.& Jous** | **Kazakhstan, Almaty** | *Atraphaxis spinosa.* |
|  | **2258** | **Coeur.& Jous** | **Kazakhstan, Almaty, Shelik** | *Atraphaxis spinosa* |
| ***B.sp2 (Thuleaphis)*** | **2580** | **Coeur.& Jous** | **Kazakhstan, Ongtustik,** | ***Rumex tianshanicus*** |
| **Outgroups** |  |  |  |  |
| ***Aphidura bozkhoae*** | **2223** | **Coeur.& Jous.** | **Kazakhstan, Ongtustik, Zhabagly** | ***Prunus tianshanica*** |
| ***Anuraphis pyrilaseri*** | **2024** | **Coeur.& Jous.** | **Sicily, Catania,** **Fiumefreddo di Sicilia** | **Apiaceae sp.** |
| *Myzus persicae* | 1956 | Coeur.& Jous. | Sicily, Catania | Scrophulariaceae |
